# Impact of an educational programme on Alzheimer’s disease patients’ quality of life: results of the randomized controlled trial THERAD

**DOI:** 10.1186/s13195-021-00896-3

**Published:** 2021-09-12

**Authors:** Hélène Villars, Christelle Cantet, Eva de Peretti, Amelie Perrin, Maria Soto-martin, Virginie Gardette

**Affiliations:** 1 Geriatric Department, Toulouse University Hospital, Hopital La Grave- Cité de la Santé Place Lange TSA 60 033, 31059 Toulouse Cedex 9, France; 2 Inserm UMR 1295: Center for Research in Population Health (CERPOP) - Department of Epidemiology and Public Health, University of Toulouse, II F-31073, 37, allées Jules Guesde, 31073 Toulouse cedex, France

**Keywords:** Alzheimer’s disease, Quality of life, Educational intervention, Caregiver, Dyadic approach, Inclusive approach

## Abstract

**Background:**

Although educational interventions are recommended in Alzheimer’s disease (AD), studies assessing the impact of interventions such as “therapeutic patient education” are scarce. Indeed, the intrinsic nature of the disease is considered a barrier to patients’ involvement in such approaches. We aimed to evaluate an intervention by using a “dyadic” approach (patient and caregiver) in both intervention and assessment.

**Methods:**

THERAD is a monocentric, randomized, controlled trial assessing the effects of a 2-month educational programme in mild to moderately severe AD patients among 98 dyads (caregiver/patient) on caregiver-reported patient quality of life (QOL) at 2 months. Community-dwelling patients and their caregivers were recruited in ambulatory units of the French Toulouse University Hospital. Self-reported patient QOL, autonomy, behavioural and psychological symptoms and caregiver QOL and burden were collected at 2, 6 and 12 months. Linear mixed models were used in modified intention-to-treat populations. We also performed sensitivity analysis.

**Results:**

A total of 196 dyads were included, 98 in each group. The mean age of the patients was 82 years, 67.7% were women, diagnosed with AD (+/- cerebrovascular component) (mean MMSE =17.6), and 56.9% lived with a partner. The mean age of the caregivers was 65.7 years, and 64.6% were women (52.3% offspring/42.6% spouses), with a moderate burden (mean Zarit score = 30.9). The mean caregiver-reported patient QOL was lower than the self-reported QOL (28.61 vs. 33.96). We did not identify any significant difference in caregiver-reported patients’ QOL (*p* = 0.297) at 2 months, but there was a significant difference in self-reported patients’ QOL at 2 months (*p* = 0.0483) or 6 months (*p* = 0.0154). No significant difference was found for the secondary outcomes. The results were stable in the sensitivity analyses.

**Conclusions:**

This randomized controlled trial assessing an educational intervention in 196 dyads (Alzheimer’s disease affected patient/caregiver) highlights the need to better consider the patient’s point of view, since only the self-reported QOL was improved. Additional studies using this dyadic approach are necessary in targeted subpopulations of caregivers (spouse vs. child, gender) and of patients (severity of cognitive impairment or behavioural disturbances)

**Trial registration:**

THERAD study NCT01796314. Registered on February 19, 2013.

## Introduction

Alzheimer’s disease (AD) patients’ care and support of their family is a major issue in the health care systems of Western countries [1]. By affecting one’s cognition, emotional processes and behaviour, AD modifies the nature of the relationship between the person and his or her caregiver, usually a relative, and consequently the role of each individual in the family and social sphere [2]. Even if this change is sometimes positive for the relationship, it can lead to what has been called a “burden”, which, shouldered by informal caregivers, has been reported in the literature as causing poor physical and mental health (depression, cardiovascular disease, anxiety) [3].

Unfortunately, there is currently a lack of safe and sufficiently effective pharmacological treatment to alleviate AD symptoms and their consequences on family life, leading to nonpharmacological therapies being placed at the forefront of therapeutic strategies [4].

Among the variety of nonpharmacological interventions designed to meet the complex needs of this population, despite not always being tested in high-quality trials, “psychoeducational approaches” have become increasingly popular over the last two decades [5]. Several types of psychoeducational strategies have been developed in AD, mostly offered either to caregivers or patients but, only recently, to the dyad (patient/caregiver). They mainly belong to multicomponent interventions, including a formalized educational programme and/or psychological support and/or respite and/or pharmacological treatment [6, 7]. The literature has reported positive effects for these multicomponent interventions, including psychoeducational interventions on caregivers’ outcomes, such as knowledge and feelings of competence [8], depression [6], physical and mental health [9], anxiety [10], well-being and quality of life [11] and burden [7, 12, 13], as well as on the patients’ behavioural and psychological symptoms of dementia (BPSD) [14], well-being [15] and admission to long-stay care [16]. Negative results were reported for the patients’ mood or cognition [17] and autonomy [18].

However, no study has evaluated an educational programme in isolation. The two interventions with the most “educational” content (not purely due to the inclusion of caregiver support) are DAISY [19] and AIDMA [18] but did not show any efficacy on patient outcomes: cognition [19] autonomy [18], quality of life (QOL) or behaviour [18, 19], or on the caregivers’ depression, burden or QOL despite improvements in the “sense of competence” of the caregiver [19].

The most effective model of “educational intervention”, called therapeutic patient education (TPE), is a “tailored person-centred lasting component of patient management”, recommended for use with 60 diseases by the World Health Organization (WHO) and shown to be effective in many chronic conditions [20]. TPE is recommended in AD routine care [21], but there is currently a need for additional evidence about the most relevant manner to implement it. TPE aims to develop disease awareness and skills for self-management behaviours through validated tools delivered by specifically trained health professionals. TPE can induce self-management behaviours and changes in patients’ lifestyles in many chronic conditions (e.g. self-monitoring of asthma [22], adherence to medication in HIV/AIDS [23], health behaviours in general in cardiac rehabilitation [24]). However, in AD, the intrinsic characteristics of the disease (cognitive impairment and anosognosia) have long been considered a real barrier to TPE implementation by AD patients themselves. Indeed, the patient’s ability to acquire new skills has been assumed to be so impaired that it constitutes an obstacle to his or her involvement. For this reason, the caregiver, who bears a substantial burden, appeared first as the “real beneficiary” of any TPE approach in AD and the targeted population of TPE programmes in trials [25]. Nevertheless, psychoeducational approaches targeting caregivers (or both patients and caregivers) can potentially modify the caregiver’s attitude, as demonstrated in paediatric care [26] and psychiatric care [27] to the benefit of the patient [27]. Thus, in light of these data, we assumed that TPE could induce a change in the caregiver’s attitudes towards the AD patient, secondarily leading to a positive impact on the patient’s quality of life. We therefore designed a trial assessing the impact of a TPE programme on AD patients’ QOL from a “dyadic” perspective (caregiver/patient) [28] in both intervention and assessment by considering the “dyad” as the true beneficiary.

## Methods

### Study design

THERAD (Therapeutic Education in Alzheimer’s Disease NCT01796314 in clinicaltrials.gov) is a monocentric, randomized, single blind, controlled trial assessing TPE in AD. Investigators and raters were blinded to group allocation. *Details of the study protocol have been* published previously [29].

### Participants

In total, 196 dyads (patient/caregiver) were recruited. Patients were community-dwelling AD patients of all ages suffering from mild to moderately severe AD (Mini-Mental State Examination (MMSE) [30] score 11–26), with or without a cerebrovascular component, receiving support from a family caregiver (nonprofessional family member living with the patient or providing support at least 3 times a week or 8 h a week) were eligible for inclusion. The AD diagnosis was based on DSM-IV criteria, imaging (magnetic resonance imaging or computed tomography scan) and biology. Recruitment was performed in the memory clinic and geriatric units of the Toulouse University Hospital (TUH) between 1 January 2013 and 31 December 2015.

We conducted a sample size estimation based on the existing literature [31, 32] and a pilot study we previously carried out [33]. The size was initially 170 dyads; however, during data monitoring, an unexpected 11% of unexploitable data for the primary outcome led us to include 26 additional dyads. This methodological choice appeared to be important for maintaining the statistical power of the results.

### Intervention

**Fig. 1:**
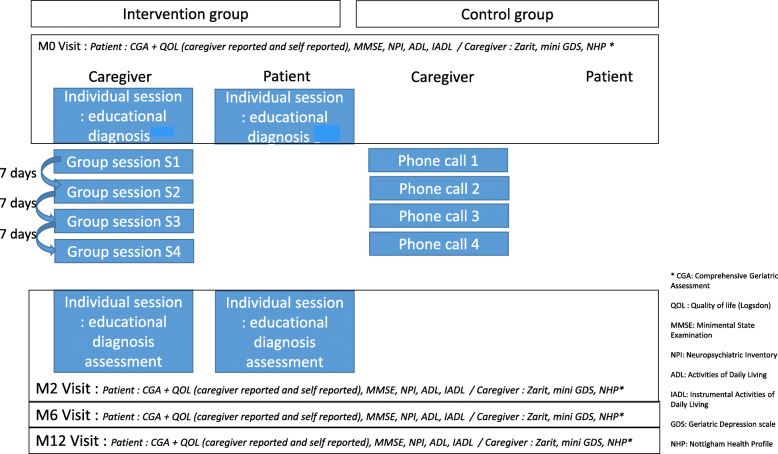
Design of the THERAD study intervention

The intervention consisted of a TPE programme of 2 months duration, as described in Fig. 1.

The intervention involved two individual sessions for patients and two for caregivers: at baseline (M0) and after 2 months (M2)). Additionally, the caregivers received four weekly group sessions (S1- S4) between M0 and M2. Individual and group sessions were conducted in the geriatric department of the TUH in a dedicated room of the ambulatory unit.

In the intervention group, each member of the dyad underwent a baseline “educational diagnosis”, the first step of TPE. The patient was questioned by semidirective interviews on his or her representations and beliefs about AD, life history, needs and requests. In a more open interview, the caregiver was questioned about his or her feelings and concerns, which helped in formulating a meaningful project for the dyad involving reachable goals and identifying skills to be acquired or strengthened. The individual session for the caregiver was 45 min, while the patient’s session was more variable between individuals (from 15 to 45 min).

The four weekly group sessions for caregivers were 3 h long and performed in small groups of six caregivers by multidisciplinary trained health professionals. Each session aimed to develop the caregivers’ understanding of their relatives’ illness (knowledge about the disease, crisis management of distressing or disruptive BPSD) and coping strategies (e.g. to adapt the communication style in stressful situations, strategies to find resources and a general understanding of care pathways) [29, 33]. The content used pedagogic methods and tools (storytelling, brainstorming, drawings, videos, quizzes) [34] designed to be reproducible. Each collective session was provided by the same professionals: a geriatrician and a nurse (S1 and S2) (a pharmacist also designed S2), a nurse and psychologist (S3) and a nurse and social worker (S4). The focus for the patient was the increase in their well-being in the daily caregiving relationship.

Last, just before the M2 visit, each member of the dyad benefitted from their second individual session to reformulate their objectives and classify them as “achieved” or “to reach”. Additional advice was delivered, and satisfaction was collected from a questionnaire completed by both patients and caregivers. No joint session had been implemented.

The control group was designed as an “attention control group” (participants receiving social attention as subjects in the intervention group—but no other elements of the intervention). Indeed, participants benefited from routine medical care, and caregivers received phone calls of a short duration (5 min) as a control condition, comprised of nonspecific and open-ended questions (“Did any change in your situation happen?” “How are you? How is the condition of your relative?”) They were delivered weekly to have the same frequency of interactions with our team as subjects in the intervention group.

### Outcomes

The primary outcome measure was a change in the AD patient’s QOL at 2 months, on the Logsdon QOL-AD scale and rated by the primary caregiver [35]. The Logsdon QOL-AD is a 13-item questionnaire that uses a 4-point Likert scale, with scores in the range 0–52 points. This scale has been validated both for self-reporting (if MMSE scores ≥ 11) and proxy (caregiver) reporting. We also collected the self-assessed Logsdon QOL-AD scores as a secondary outcome at 2, 6 and 12 months.

Several secondary endpoints were assessed at 2, 6 and 12 months: patient’s BPSD based on the Neuro Psychiatric Inventory (NPI) [36] and functional autonomy based on the Activities of Daily Living scale (ADL) [37] and the Instrumental Activities of Daily Living scale (IADL) [38]; caregiver’s burden through the Zarit Burden Inventory (ZBI) [39] and QOL based on the Nottingham Health Profile (NHP) [40].

We also collected several variables known to impact the patient’s QOL [41]: caregiver’s mood based on the mini Geriatric Depression Scale (GDS) [42] and patients’ MMSE score [30]. Finally, patient and caregiver satisfaction data were collected from a questionnaire completed at 2 months.

### Statistical analysis

Baseline characteristics of the participants who were included in the intention-to-treat (ITT) population (i.e. including all randomly assigned participants) are presented as the mean and standard deviation (SD) for quantitative variables and as frequency and percentage for qualitative variables. For some scales (Logsdon QOL-AD, MMSE, ZBI), rare missing items were imputed up to 10% of the total number of items, or, as proposed by Logsdon [35] in the case of one or two missing items for the Logsdon QOL-AD scale; otherwise, the score was considered missing. The main imputation method used was the mean score of the remaining items, except for the NHP for which the proportionality rule was applied.

For continuous outcomes, linear mixed models, adjusted by the baseline data to take into account the regression to the mean [43], were used to assess the effect of the intervention (the mixed procedure from SAS). Analyses were performed on a modified ITT (mITT) population (i.e. including all randomly assigned participants with outcomes measured at baseline and with at least one post-baseline visit). For the binary outcomes, logistic mixed models in the ITT population, with the baseline value included in the dependent variable, were used (the Glimmix procedure from SAS).

For each mixed model, we included the following fixed effects: baseline value (only for continuous outcomes), intervention group, time as a continuous variable, and interaction between group and time. The mixed models included subject-specific random effects to take into account the intrasubject correlation: a random intercept to take into account the heterogeneity of the outcome at the first timepoint and a random slope (if significant) to take into account the heterogeneity of the slopes between subjects.

Subgroup analyses were performed to study the effect of the intervention according to the level of cognitive function (MMSE) and caregiver burden (ZBI) using linear mixed models as described above.

Two sensitivity analyses were performed for the Logsdon QOL score. The first analysis was conducted in the per-protocol population, excluding major protocol violations (poor compliance). A good TPE observance was defined by participation in 2 individual sessions and at least three or four collective sessions. The second one was performed with linear mixed models, including the baseline value in the dependent variable to include all randomized subjects and to model the trajectory with additional time. SAS version 9.4 (Cary, NC, USA) software was used for all analyses, and the statistical significance was set as 5%.

## Results

**Fig. 2:**
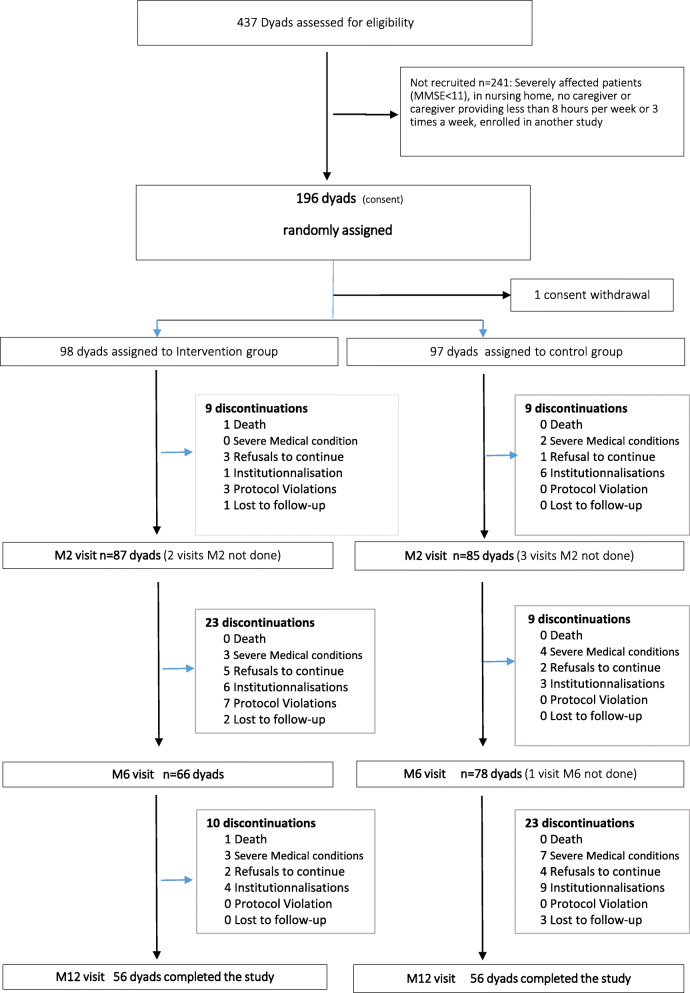
Flowchart of the THERAD study population

A total of 196 dyads were included; 172 (87.8%) were followed until 2 months, and 112 (57.2%) completed the 12-month visit (Fig. 2). The baseline characteristics of the dyads are presented in Table 1. With regard to the patients, their mean age was 82 years, 67.7% were women, 16.9% had a bachelor degree or higher, 56.9% lived with a partner, they were diagnosed with AD (or AD with a cerebrovascular component) for a mean time of 15 months, their mean MMSE was 17.65, 48.2% had at least one incapacity of ADL, and their NPI mean score was 21.7 and 49.2% were being treated with antidementia drugs. The mean age of caregivers was 65.7 years, 64.6% were women, they were mainly a close relative (52.3% were offspring, 42.6% were spouses), living at home with the patient (53.3% spending a mean time of 21.67 h per week in caregiving) with a moderate burden (mean ZBI score = 30.9) and a low QOL (mean NHP score = 119.60). These characteristics were well balanced between the groups, with little heterogeneity in the patients’ comorbidities, dementia aetiology, time elapsed since diagnosis, two NPI items (hallucinations, apathy) or speech therapy.

**Table 1:** Baseline dyads’ characteristics

A. Patients’ characteristics*	Total population ***n*** = 195 Mean (SD) or *n* (%)	Group	
		Intervention ***n*** = 98 Mean (SD) or *n* (%)	Control ***n*** = 97 Mean (SD) or *n* (%)
**Sociodemographic data**			
**Age (years)**	82.03 (5.88)	81.94 ( 6.38)	82.12 (5.36)
1: < 75 years	22 (11.28%)	81.94 (5,88)	82.12 (5.36)
2: [75–85[ years	108 (55.38%)	13 (13.27%)	9 (9.28%)
3: ≥ 85 years	65 (33.33%)	49 (50.00%)	59 (60.82%)
**Women**	132 (67.69%)	66 (67.35%)	66 (68.04%)
**Educational level**			
Elementary or no formal	51 (26.15%)	20 (20.41%)	31 (31.96%)
Primary school certificate or less	60 (30.77%)	36 (36.73%)	24(24.74%)
Secondary education/high school	51 (26.15%)	25 (25.51%)	26 (26.80%)
Bachelor’s degree and higher	33 (16.92%)	17 (17.35%)	16 (16.49%)
**Professional activity in the past**	173 (88.72%)	88 (89.80%)	85 (87.63%)
**Living in a marital status**	111 (56.92%)	56 (57.14%)	55 (56.70%)
**Alzheimer’s disease**	161 (82.56%)	87 (88.78%)	74 (76.29%)
**Mixed dementia (AD and vascular)**	34 (17.44%)	11 (11.22%)	23 (23.71%)
**MMSE mean (SD)**	17.65 (4.11)	18.12 (4.04)	17.16 (4.14)
**Stage of severity (MMSE)**			
[21–26]	51 (26.15%)	30 (30.61%)	21 (21.65%)
[15;20]	77 (39.49%)	38 (38.78%)	39 (40.21%)
[11–15]	67 (34.36%)	30 (30.61%)	37 (38.14%)
**Time elapsed since diagnosis (months), (*n*= 176)**	14.57 (19.25)	12.44 (18.97)	16.80 (19.39)
**Comorbidities CIRS-G**	9.91 (3.85)	9.30 (3.82)	10.53 (3.80)
**Functional autonomy**			
ADL	5.33 (0.89)	5.42 (0.79)	5.23 (0.97)
Score < 6/6 (at least one incapacity)	101 (51.79%)	46 (46.94%)	55 (56.70%)
IADL (*n* = 193)	1.61 (1.23)	1.63 (1.24)	(1.22)
Score 0–1 (≥ 4 incapacities)	102 (52.85%)	52 (54.17%)	50 (51.55%)
**Gait and balance one leg balance < 5 s (*n*** **= 184)**	121 (65.76%)	60 (64.52%)	61 (67.03%)
**Visual impairment**	111 (56.92%)	56 (57.14%)	55 (56.70%)
**Hearing impairment**	40 (20.51%)	17 (17.35%)	23 (23.71%)
**Quality of life hetero-assessed by caregivers (*n*** **= 185)**	28.61 (5.24)	29.33 (5.12)	27.84 (5.27)
**Quality of life self-assessed by patients (*n*** **= 145)**	33.93 (6.03)	33.66 (5.53)	34.24 (6.59)
**Behavioural and psychological symptoms**			
NPI total score (*n* = 178)	21.77 (18.40)	22.25 (18.82)	21.26 (18.05)
Delusions	43 (22.05%)	17 (17.35%)	26 (26.80%)
Hallucinations (*n* = 194)	38 (19.59%)	13 (13.40%)	25 (25.77%)
Agitation or aggressiveness (*n* = 193)	105 (54.40%)	50 (51.02%)	55 (57.89%)
Depression/dysphoria (*n* = 194)	116 (59.79%)	59 (60.82%)	57 (58.76%)
Anxiety (*n* = 194)	140 (72.16%)	74 (75.51%)	66 (68.75%)
Euphoria	41 (21.03%)	23 (23.47%)	18 (18.56%)
Apathy	115 (58.97%)	51 (52.04%)	64 (65.98%)
Disinhibition, (*n* = 190)	51 (26.84%)	20 (20.83%)	31 (32.98%)
Irritability (*n* = 194)	125 (64.43%)	61 (62.24%)	64 (66.67%)
Aberrant motor activity (*n* = 194)	40 (20.62%)	21 (21.43%)	19 (19.79%)
Sleep disorders (*n* = 192)	73 (38.02%)	35 (36.08%)	38 (40.00%)
Eating disorders (*n* = 192)	76 (39.58%)	37 (38.54%)	39 (40.63%)
**Pharmacological therapies**			
Acetylcholinesterase inhibitors or NMDA receptor blocker	96 (49.23%)	43 (43.88%)	53 (54.64%)
Psychotropes	46 (23.59%)	25 (25.51%)	21 (21.65%)
Antipsychotic drug	5 (2.56%)	1 (1.02%)	4 (4.12%)
Anxiolytics	19 (9.74%)	8 (8.16%)	11 (11.34%)
Sedative	8 (4.10%)	7 (7.14%)	1 (1.03%)
Antidepressant therapy	26 (13.33%)	14 (14.29%)	12 (12.37%)
**Nonpharmacological therapies**			
Physical therapist	27 (13.85%)	13 (13.27%)	14 (14.43%)
Ergotherapist	4 (2.05%)	2 (2.04%)	2 (2.06%)
Psychologist	1 (0.51%)	0 (0.00%)	1 (1.03%)
Speech therapist	24 (12.31%)	19 (19.39%)	5 (5.15%)
Day care centre	12 (6.15%)	6 (6.12%)	6 (6.19%)
Home help (daily living activities)	38 (19.49%)	18 (18.37%)	20 (20.62%)
Domestic help (cleaning)	23 (11.79%)	15 (15.31%)	8 (8.25%)
Nurse	58 (29.74%)	26 (26.53%)	32 (32.99%)
Specialized nurse	6 (3.08%)	3 (3.06%)	3 (3.09%)
Home meal deliveries	2 (1.03%)	1 (1.02%)	1 (1.03%)
B. Caregivers’ characteristics ^a^	**Total population** ***n*= 195**	**Group**	
		**Intervention** ***n*= 98**	**Control** ***n*= 97**
**Sociodemographic data**			
**Age (years)**	65.75 (12.62)	66.13 (12.59)	65.36 (12.69)
**≤ 65**	106 (54.36%)	49 (50.00%)	57 (58.76%)
> 65	89 (45.64%)	49 (50.00%)	40 (41.24%)
**Gender = women**	126 (64.62%)	60 (61.22%)	66 (68.04%)
**Educational level, (*n*= 192 )**			
Primary school certificate or less	29 (15.10%)	14 (14.43%)	15 (15.79%)
Secondary education/high school	60 (31.25%)	30 (30.93%)	30 (31.58%)
Bachelor’s degree and higher	103 (53.65%)	53 (54.64%)	50 (52.63%)
**Professionally active (or in the past) (*n*** **= 191)**	179 (93.72%)	92 (94.85%)	87 (92.55%)
**Caregiver status**			
Child	102 (52.31%)	48 (48.98%)	54 (55.67%)
Spouse	83 (42.56%)	44 (44.90%)	39 (40.21%)
Brother or sister	1 (0.51%)	0 (0.00%)	1 (1.03%)
Nephew/niece	3 (1.54%)	1 (1.02%)	2 (2.06%)
Daughter-in-law or son-in-law	6 (3.08%)	5 (5.10%)	1 (1.03%)
**Living in a marital status**	160 (82.05%)	85 (86.73%)	75 (77.32%)
**Living arrangement**			
Caregiver living at home with the patient	104 (53.33%)	54 (55.10%)	50 (51.55%)
Patient and caregiver living apart	91 (46.67%)	44 (44.90%)	47 (48.45%)
< 6.21 miles	53 (27.18%)	25 (25.51%)	28 (28.87%)
> 6.21 miles	38 (19.49%)	19 (19.39%)	19 (19.59%)
**Length of caregiving (*n*= 193) years**			
1: < 1 year	53 (27.46%)	26 (26.80%)	27 (28.13%)
2: between 1 and 3 years	84 (43.52%)	42 (43.30%)	42 (43.75%)
3: > 3 years	56 (29.02%)	29 (29.90%)	27 (28.13%)
**Hours of caregiving per week**	21.67 (13.66)	22.68 (15.19)	20.65 (11.91)
**Medical chronic condition**	67 (34.36%)	38 (38.78%)	29 (29.90%)
**Level of exhaustion and burden (*n*= 194)**			
**Zarit score (*n*** **= 194) mean**	30.89 (15.77)	29.97 (16.19)	31.83 (15.36)
**1: [0–20]**	58 (29.90%)	41 (41.84%)	40 (41.67%)
**2: [20–40]**	81 (41.75%)	24 (24.49%)	31 (32.29%)
**3: > 40**	55 (28.35%)	55 (56.70%)	58 (59.79%)
**Quality of life (NHP score)**	119.60 (112.00)	119.18 (108.65)	120.02 (115.78)

^a^The population size is presented in brackets in case of missing data (*n* < 195)

Regarding compliance, 87 patients (88.8%) and 74 caregivers (75.5%) showed good TPE observance.

The estimated changes in outcomes from baseline are presented in Table 2 and detailed below.

**Table 2:** Effect of the THERAD intervention vs control on primary and secondary outcomes: change from baseline to 2, 6 and 12 months (using linear mixed models in modified intention-to-treat population for continuous outcomes, and logistic mixed models in intention-to-treat population for binary outcomes)

	Estimated change from baseline mean^**a**^ or OR^b^ (95%CI)		Estimated differences in change from baseline mean or OR^b^ (95%CI) ***p*** value	Estimated change from baseline mean^**a**^ or OR^b^ (95%CI)		Estimated differences in change from baseline mean or OR^b^ (95%CI) ***p*** value	Estimated change from baseline mean^**a**^ or OR^b^ (95%CI)		Estimated differences in change from baseline mean or OR^b^ (95%CI) ***p*** value
Outcome	Intervention	Control	Intervention vs Control	Intervention	Control	Intervention vs Control	Intervention	Control	Intervention vs Control
	M2–M0			M6–M0			M12–M0		
QOL patient by caregiver	0.77 [− 0.13 to 1.66]	0.09 [− 0.83 to 1.00]	0.68 [− 0.60 to 1.96] *p =* 0.2970	0.15 [− 0.65 to 0.94]	− 0.18 [− 0.98 to 0.62]	0.33 [− 0.80 to 1.46] *p =* 0.5651	− 0.78 [− 1.93 to 0.38]	− 0.59 [− 1.78 to 0.61]	− 0.19 [− 1.85 to 1.47] *p =* 0.8198
QOL patient by patient	0.72 [− 0.44 to 1.88]	− 0.98 [− 2.19 to 0.24]	1.70 [0.01 to 3.38] ***p =*0.0483**	0.96 [0.00 to 1.91]	− 0.78 [− 1.80 to 0.23]	1.74 [0.34 to 3.15] ***p*= 0.0154**	1.32 [0.07 to 2.56]	− 0.50 [− 1.89 to 0.89]	1.82 [− 0.06 to 3.69] *p =* 0.0575
NPI f*g	− 2.52 [− 6.32 to 1.29]	0.26 [− 3.51 to 4.03]	− 2.77 [− 8.13 to 2.59] *p =* 0.3090	− 2.64 [− 5.90 to 0.61]	1.09 [− 2.18 to 4.35]	− 3.73 [− 8.34 to 0.89] *p* = 0.1126	− 2.83 [− 6.89 to 1.23]	2.33 [− 1.94 to 6.60]	− 5.16 [− 11.05 to 0.73] *p =* 0.0859
ADL	− 0.09 [− 0.20 to 0.02]	− 0.15 [− 0.25 to − 0.04]	0.06 [− 0.09 to 0.21] *p =* 0.4519	− 0.23 [− 0.32 to − 0.13]	− 0.24 [− 0.33 to − 0.15]	0.02 [− 0.12 to 0.15] *p =* 0.8150	− 0.43 [− 0.59 to − 0.27]	− 0.38 [− 0.54 to − 0.23]	− 0.05 [− 0.27 to 0.18] *p =* 0.6855
IADL (≥ 4 vs < 4 incapacities)	1.60 [1.10 to 2.31]	1.46 [1.04 to 2.06]	1.09 [0.69 to 1.72] *p =* 0.7096	4.07 [1.34 to 12.31]	3.14 [1.13 to 8.69]	1.30 [0.33 to 5.11] *p =* 0.7096	16.53 [1.80 to 151.63]	9.83 [1.28 to 75.52]	1.68 [0.11 to 26.12] *p =* 0.7096
MMSE	− 0.38 [− 0.95 to 0.19]	− 0.76 [− 1.32 to − 0.19]	0.38 [− 0.43 to 1.18] *p* = 0.3560	− 1.08 [− 1.63 to − 0.52]	− 1.07 [− 1.61 to − 0.53]	− 0.01 [− 0.79 to 0.77] *p* = 0.9825	− 2.12 [− 2.92 to − 1.32]	− 1.53 [− 2.31 to − 0.76]	− 0.59 [− 1.71 to 0.53] *p* = 0.3007
One leg balance (abnormal vs normal)	1.00 [0.87 to 1.14]	1.08 [0.93 to 1.25]	0.92 [0.76 to 1.13] *p* = 0.4282	0.99 [0.65 to 1.50]	1.26 [0.82 to 1.94]	0.79 [0.43 to 1.43] *p* = 0.4282	0.98 [0.42 to 2.25]	1.58 [0.67 to 3.76]	0.62 [0.19 to 2.04] *p =* 0.4282
Zarit	− 2.38 [− 4.50 to − 0.25]	− 0.14 [− 2.24 to 1.96]	− 2.24 [− 5.23 to 0.75] *p* = 0.1411	− 1.19 [− 3.18 to 0.80]	0.35 [− 1.62 to 2.31]	− 1.54 [− 4.33 to 1.26] *p* = 0.2786	0.58 [− 2.30 to 3.46]	1.07 [− 1.79 to 3.92]	− 0.49 [− 4.54 to 3.57] *p =* 0.8128
Mini-GDS (≥ 1 vs =0)	1.02 [0.86 to 1.22]	0.89 [0.74 to 1.06]	1.15 [0.89 to 1.48] *p =* 0.2759	1.06 [0.63 to 1.80]	0.70 [0.41 to 1.19]	1.52 [0.71 to 3.22] *p* = 0.2759	1.13 [0.39 to 3.26]	0.49 [0.17 to 1.43]	2.30 [0.51 to 10.39] *p =* 0.2759
NHP	− 0.59 [− 14.48 to 13.30]	− 6.38 [− 20.02 to 7.27]	5.79 [− 13.68 to 25.26] *p* = 0.5582	2.57 [− 11.03 to 16.17]	1.95 [− 11.34 to 15.23]	0.62 [− 18.39 to 19.64] *p* = 0.9484	7.31 [− 12.99 to 27.60]	14.43 [− 5.37 to 34.23]	− 7.12 [− 35.48 to 21.23] *p =* 0.6199

^a^Mean (95%CI): estimated with the mean values at baseline

^b^Odds ratio (95%CI)

### Quality of life caregiver-reported (Fig. 3A)

At 2 months, the change from baseline in the patient’s QOL reported by the caregiver was 0.77 (95% CI [0.13, 1.66]) for the intervention group and 0.09 (95% CI [− 0.83, − 1.00]) for the control group, representing a nonsignificant 0.68-point difference (95% CI [− 0.60, 1.95]; *p* = 0.297) between groups.

**Fig. 3:**
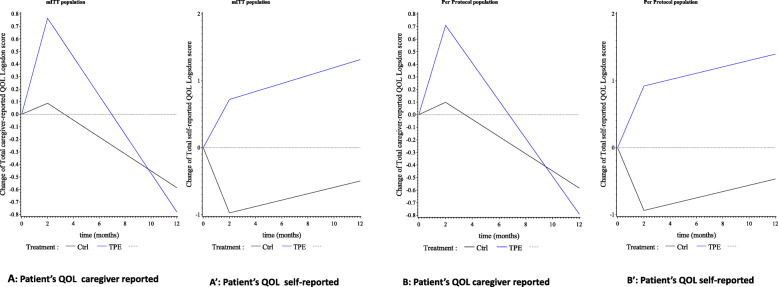
Change from baseline over time in patients’ QOL caregiver-reported and self-reported by patients in the mITT (*n* = 155) (**A**, **A’**) and PP (*n* = 148) (**B**, **B’**) populations

The difference in QOL observed at 6 and 12 months decreased to 0.33 and − 0.19, respectively, but was not statistically significant.

### Quality of life self-reported by the patient (Fig. 3A’)

At 2 months, the self-reported patients’ QOL increased by 0.72 (95% CI [− 0.44, 1.88]) for the intervention group and decreased by − 0.98 (95% CI [− 2.19, 0.24]) for the control group, representing a significant 1.70 point difference (95% CI [0.01, 3.38]) in favour of TPE (*p* = 0.0483), which was sustained at 6 months (1.74, 95% CI [0.34, 3.1]; *p* = 0.0154) but of borderline significance at 12 months (*p* = 0.0575).

No statistically significant difference was found for the other secondary outcomes.

#### Sensitivity analyses

The per protocol analyses produced stable conclusions (Table 3). The effect observed for self-reported QOL was enhanced (Fig. 3B, B’).

**Table 3:** Effect of the THERAD intervention vs control on patient’s QOL caregiver-reported and self-reported: sensitivity analysis in per protocol population and/or with another mixed linear model method

	Estimated change from baseline mean^a^ (95%CI)		Estimated differences in change from baseline mean (95%CI) ***p*** value	Estimated change from baseline mean^a^ (95%CI)		Estimated differences in change from baseline mean (95%CI) ***p*** value	Estimated change from baseline mean^a^ (95%CI)		Estimated differences in change from baseline mean (95%CI) ***p*** value
Outcome	Intervention	Control	Intervention vs Control	Intervention	Control	Intervention vs Control	Intervention	Control	Intervention vs Control
	M2–M0			M6–M0			M12–M0		
**Per-protocol population model 1**									
QOL patient by caregiver (int: 1 = 72, ctrl: *n* = 76)	0.71 [− 0.23 to 1.65]	0.10 [− 0.82 to 1.01]	0.61 [− 0.70 to 1.93] *p =* 0.3595	0.11 [− 0.72 to 0.94]	− 0.17 [− 0.98 to 0.63]	0.28 [− 0.88 to 1.45] *p =* 0.6287	− 0.79 [− 1.96 to 0.38]	− 0.58 [− 1.78 to 0.61]	− 0.21 [− 1.88 to 1.47] *p =* 0.8086
QOL patient by patient (int: *n* = 56, ctrl: *n* = 53)	0.92 [− 0.30 to 2.14]	− 0.94 [− 2.17 to 0.29]	1.86 [0.13 to 3.60] ***p =*0.0356**	1.11 [0.11 to 2.11]	− 0.75 [− 1.78 to 0.28]	1.86 [0.42 to 3.31] ***p =*0.0118**	1.40 [0.14 to 2.66]	− 0.47 [− 1.87 to 0.93]	1.87 [− 0.02 to 3.76] *p =* 0.0529
**Per-protocol population model 2**									
QOL patient by caregiver (int: *n* = 73, ctrl: *n* = 90)	− 0.18 [− 0.38 to 0.02]	− 0.06 [− 0.26 to 0.15]	− 0.12 [− 0.41 to 0.16] *p =* 0.4023	− 0.53 [− 1.14 to 0.07]	− 0.17 [− 0.78 to 0.44]	− 0.36 [− 1.22 to 0.49] *p =* 0.4023	− 1.07 [− 2.27 to 0.14]	− 0.34 [− 1.56 to 0.88]	− 0.73 [− 2.45 to 0.99] *p =* 0.4023
QOL patient by patient (int: *n* = 61, ctrl: *n* = 67)	0.21 [− 0.01 to 0.43]	− 0.06 [− 0.31 to 0.18]	0.28 [− 0.05 to 0.60] *p =* 0.0953	0.64 [− 0.02 to 1.29]	− 0.19 [− 0.92 to 0.53]	0.83 [− 0.15 to 1.81] *p =* 0.0953	1.27 [− 0.04 to 2.59]	− 0.39 [− 1.84 to 1.06]	1.66 [− 0.29 to 3.62] *p =* 0.0953
**ITT population model 2**									
QOL patient by caregiver (int: *n* = 95, ctrl: *n* = 90)	− 0.18 [− 0.37 to 0.02]	− 0.06 [− 0.26 to 0.15]	− 0.12 [− 0.40 to 0.16] *p =* 0.4005	− 0.53 [− 1.12 to 0.06]	− 0.17 [− 0.78 to 0.44]	− 0.36 [− 1.21 to 0.49] *p =* 0.4005	− 1.06 [− 2.24 to 0.12]	− 0.33 [− 1.55 to 0.88]	− 0.72 [− 2.42 to 0.97] *p =* 0.4005
QOL patient by patient (int: *n* = 78, ctrl: *n* = 67)	0.22 [0.00 to 0.43]	− 0.06 [− 0.31 to 0.18]	0.28 [− 0.04 to 0.61] *p =* 0.0858	0.65 [0.01 to 1.30]	− 0.19 [− 0.92 to 0.53]	0.85 [− 0.12 to 1.82] *p =* 0.0858	1.31 [0.02 to 2.60]	− 0.39 [− 1.84 to 1.06]	1.70 [− 0.24 to 3.63] *p =* 0.0858

Model 1: linear mixed model adjusted on baseline data

Model 2: linear mixed model with baseline value included in the dependent variable

^a^ Mean (95%CI): estimated with the mean values at baseline for model 1

#### Subgroup analyses

Table 4 presents the results of caregiver-reported QOL and self-reported QOL in the subgroup analysis.

**Table 4:** Patient’s QOL caregiver-reported and self-reported in subgroup analysis of the THERAD study (results from linear mixed models in modified intention-to-treat population). ^1^Mean (95%CI): estimated with the mean values at baseline

		Estimated change from baseline mean^1^ (95%CI)		Estimated differences in change from baseline mean (95%CI) ***p*** value	Estimated change from baseline mean^1^ (95%CI)		Estimated differences in change from baseline mean (95%CI) ***p*** value	Estimated change from baseline mean^1^ (95%CI)		Estimated differences in change from baseline mean (95%CI) ***p*** value
		Intervention	Control	Intervention vs Control	Intervention	Control	Intervention vs Control	Intervention	Control	Intervention vs Control
Outcome	Subgroup	M2–M0			M6–M0			M12–M0		
Patient’s QOL caregiver-reported	1. tot Zarit: > 40	0.33 [− 1.48 to 2.14]	− 0.74 [− 2.40 to 0.92]	1.07 [− 1.32 to 3.45] *p =* 0.3783	0.92 [− 0.71 to 2.56]	− 1.02 [− 2.48 to 0.44]	1.95 [− 0.17 to 4.06] *p =* 0.0709	1.82 [− 0.70 to 4.34]	− 1.45 [− 3.66 to 0.76]	3.27 [− 0.03 to 6.57] *p =* 0.0523
Patient’s QOL caregiver-reported	2. tot Zarit: [20;40]	1.31 [− 0.09 to 2.70]	− 0.34 [− 1.72 to 1.03]	1.65 [− 0.31 to 3.61] *p =* 0.0985	0.21 [− 0.99 to 1.41]	− 0.61 [− 1.81 to 0.60]	0.82 [− 0.88 to 2.51] *p =* 0.3438	− 1.44 [− 3.06 to 0.19]	− 1.00 [− 2.75 to 0.75]	− 0.44 [− 2.83 to 1.95] *p =* 0.7185
Patient’s QOL caregiver-reported	3. tot Zarit: ≤ 20	0.45 [− 1.10 to 2.00]	2.00 [0.17 to 3.84]	− 1.55 [− 3.88 to 0.78] *p =* 0.1898	− 0.20 [− 1.61 to 1.20]	1.58 [0.00 to 3.16]	− 1.78 [− 3.81 to 0.24] *p =* 0.0835	− 1.18 [− 3.17 to 0.80]	0.94 [− 1.24 to 3.13]	− 2.13 [− 5.01 to 0.76] *p =* 0.1468
Patient’s QOL caregiver-reported	4. tot Zarit: > 40 vs ≤ 20			2.62 [− 0.71 to 5.95] *p =* 0.1226			3.73 [0.80 to 6.66] *p =*0.0129*			5.40 [1.01 to 9.79] *p =*0.0163*
Patient’s QOL caregiver-reported	5. tot Zarit: [20;40] vs ≤ 20			3.20 [0.16 to 6.24] *p =*0.0390*			2.60 [− 0.03 to 5.23] *p =* 0.0530			1.69 [− 2.05 to 5.43] *p =* 0.3719
Patient’s QOL caregiver-reported	1. tot MMS: ≤ 15	1.33 [− 0.36 to 3.02]	0.14 [− 1.44 to 1.72]	1.19 [− 1.12 to 3.50] *p =* 0.3112	0.60 [− 0.94 to 2.14]	− 0.25 [− 1.60 to 1.11]	0.85 [− 1.21 to 2.90] *p =* 0.4158	− 0.49 [− 2.78 to 1.80]	− 0.83 [− 2.86 to 1.21]	0.33 [− 2.73 to 3.40] *p =* 0.8296
Patient’s QOL caregiver-reported	2. tot MMS: [15;20]	1.08 [− 0.34 to 2.51]	0.36 [− 1.04 to 1.75]	0.73 [− 1.26 to 2.72] *p =* 0.4713	0.59 [− 0.67 to 1.84]	− 0.05 [− 1.28 to 1.18]	0.63 [− 1.12 to 2.39] *p =* 0.4781	− 0.16 [− 1.96 to 1.64]	− 0.65 [− 2.44 to 1.14]	0.49 [− 2.05 to 3.02] *p =* 0.7059
Patient’s QOL caregiver-reported	3. tot MMS: > 20	− 0.08 [− 1.62 to 1.47]	− 0.49 [− 2.39 to 1.41]	0.41 [− 2.04 to 2.86] *p =* 0.7413	− 0.72 [− 2.07 to 0.63]	− 0.33 [− 2.02 to 1.37]	− 0.39 [− 2.56 to 1.77] *p =* 0.7198	− 1.68 [− 3.62 to 0.25]	− 0.08 [− 2.59 to 2.43]	− 1.60 [− 4.77 to 1.57] *p =* 0.3190
Patient’s QOL caregiver-reported	4. tot MMS: ≤ 15 vs > 20			0.78 [− 2.62 to 4.18] *p =* 0.6518			1.24 [− 1.77 to 4.26] *p =* 0.4167			1.94 [− 2.49 to 6.36] *p =* 0.3882
Patient’s QOL caregiver-reported	5. tot MMS: [15;20] vs > 20			0.32 [− 2.86 to 3.49] *p =* 0.8435			1.03 [− 1.78 to 3.83] *p =* 0.4710			2.09 [− 1.98 to 6.16] *p =* 0.3123
Self-reported QOL	1. tot Zarit: > 40	0.56 [− 1.76 to 2.88]	− 4.13 [− 6.60 to − 1.66]	4.69 [1.30 to 8.08] *p =*0.0069*	1.33 [− 0.62 to 3.27]	− 2.99 [− 4.94 to − 1.03]	4.31 [1.54 to 7.08] *p =*0.0026*	2.47 [− 0.17 to 5.11]	− 1.27 [− 4.56 to 2.02]	3.74 [− 0.49 to 7.98] *p =* 0.0830
Self-reported QOL	2. tot Zarit: [20;40]	1.13 [− 0.69 to 2.94]	0.52 [− 1.28 to 2.31]	0.61 [− 1.94 to 3.15] *p =* 0.6383	1.22 [− 0.24 to 2.68]	0.40 [− 1.10 to 1.90]	0.82 [− 1.26 to 2.90] *p =* 0.4368	1.37 [− 0.52 to 3.26]	0.23 [− 1.74 to 2.20]	1.14 [− 1.58 to 3.86] *p =* 0.4102
Self-reported QOL	3. tot Zarit: ≤ 20	0.42 [− 1.43 to 2.28]	− 0.66 [− 2.76 to 1.44]	1.08 [− 1.70 to 3.86] *p =* 0.4428	0.51 [− 1.02 to 2.05]	− 0.56 [− 2.32 to 1.20]	1.08 [− 1.24 to 3.39] *p =* 0.3580	0.64 [− 1.35 to 2.64]	− 0.42 [− 2.73 to 1.89]	1.06 [− 1.97 to 4.10] *p =* 0.4908
Self-reported QOL	4. tot Zarit: > 40 vs ≤ 20			3.61 [− 0.77 to 7.99] *p =* 0.1058			3.24 [− 0.36 to 6.83] *p =* 0.0769			2.68 [− 2.51 to 7.86] *p =* 0.3101
Self-reported QOL	5. tot Zarit: [20;40] vs ≤ 20			− 0.48 [− 4.24 to 3.29] *p =* 0.8030			− 0.26 [− 3.36 to 2.85] *p =* 0.8705			0.08 [− 3.99 to 4.14] *p =* 0.9706
Self-reported QOL	1. tot MMS: ≤ 15	1.17 [− 1.33 to 3.67]	− 1.30 [− 4.10 to 1.49]	2.47 [− 1.27 to 6.22] *p =* 0.1947	1.52 [− 0.54 to 3.58]	− 0.60 [− 3.05 to 1.84]	2.12 [− 1.07 to 5.32] *p =* 0.1910	2.05 [− 0.79 to 4.89]	0.44 [− 3.82 to 4.71]	1.61 [− 3.52 to 6.74] *p =* 0.5381
Self-reported QOL	2. tot MMS: [15;20]	1.65 [− 0.19 to 3.48]	− 1.06 [− 2.78 to 0.67]	2.70 [0.18 to 5.23] *p =*0.0360*	1.63 [0.12 to 3.13]	− 0.92 [− 2.34 to 0.49]	2.55 [0.47 to 4.63] *p =*0.0166*	1.60 [− 0.30 to 3.49]	− 0.72 [− 2.57 to 1.13]	2.32 [− 0.34 to 4.97] *p =* 0.0872
Self-reported QOL	3. tot MMS: > 20	− 0.41 [− 2.22 to 1.40]	− 0.69 [− 2.80 to 1.42]	0.28 [− 2.50 to 3.06] *p =* 0.8432	0.04 [− 1.45 to 1.53]	− 0.55 [− 2.30 to 1.21]	0.59 [− 1.72 to 2.89] *p =* 0.6140	0.73 [− 1.26 to 2.72]	− 0.33 [− 2.69 to 2.04]	1.05 [− 2.04 to 4.15] *p =* 0.5036
Self-reported QOL	4. tot MMS: ≤ 15 vs > 20			2.19 [− 2.48 to 6.86] *p =* 0.3556			1.54 [− 2.42 to 5.49] *p =* 0.4434			0.55 [− 5.46 to 6.57] *p =* 0.8563
Self-reported QOL	5. tot MMS: [15;20] vs > 20			2.43 [− 1.32 to 6.17] *p =* 0.2029			1.96 [− 1.12 to 5.04] *p =* 0.2097			1.26 [− 2.79 to 5.31] *p =* 0.5394

We observed a significant effect of the intervention on the caregiver-reported QOL at 6 and 12 months in the subgroups of subjects with ZBI scores between > 40 and ≤ 20 (*p* = 0.0129 and *p* = 0.0163, respectively) and at 2 months (*p* = 0.0390) in the subgroups with ZBI scores between 20 and 40 and ≤ 20.

There was no difference in the self-reported patient QOL according to the level of burden (although we observed a significant positive effect of the intervention in the subgroup of subjects with ZBI score > 40 at 2 months (*p* = 0.0069) and 6 months (*p* = 0.0026)) or the MMSE scores (even though there was a significant positive effect of the intervention in the subgroup of the subjects with MMSE score 15–20 at 2 (*p* = 0.0360) and 6 months (*p* = 0.0166)).

Last, of the 73 caregivers who completed the satisfaction questionnaire at M2 (participation rate = 83.9%), 26% were satisfied (*n* = 19) and 74% were very satisfied (*n* = 54).

## Discussion

THERAD did not find any significant effect of TPE on the caregiver-reported patient’s QOL at 2 months but there was a significant effect when it was self-reported by the patient. No other significant effect on either patient or caregiver outcomes was seen. Several reasons can explain this result. First, QOL is a multidimensional relevant criterion and a key patient-centred outcome [41, 44, 45] especially in AD care [46], and when measuring the overall objectives of an educational intervention [47], measuring the QOL of persons with dementia is challenging because of the intrinsic nature of the disease: cognitive impairment, memory loss and anosognosia [48–50]. Indeed, the patient’s ability to remember the past and thus to identify changes and make choices among items on a scale is affected by memory impairment but also by a lack of insight [48] and anosognosia [43, 44]. These symptoms, which are more prevalent as the patient’s condition worsens [50], tend to increase QOL scores and lead to a stability of QOL scores over time [44]. For this reason, the validity of self-reported QOL assessments by patients with dementia is a critical issue [51]. Therefore, researchers commonly use the rating by the caregiver as a proxy of the patient’s QOL in most dementia clinical trials and this is considered reliable [52]. However, this proxy rating may introduce bias because of the influence of the caregiver’s point of view and other factors that might influence their assessment of the patient’s QOL [47, 53]. In fact, discrepancies have been previously reported between self- and proxy-reported QOL [45, 52, 54], with caregivers underestimating the patient’s QOL [45, 55, 52] especially in the cases when they are suffering from depression [52] or exhaustion [45] themselves, as well as depression [56] or BPSD [52] in their relative suffering from AD. However, acknowledging these discrepancies and the potential bias of proxy reporting, we made the choice to use proxy-reported QOL and to also consider self-reported QOL because it was demonstrated to be complementary [52, 53, 57] and feasible by the QOL Logsdon scale. Indeed, scales were numerous [51], but the QOL-AD of Logsdon validated for both patient and caregiver use [35] was considered, and still is, as having valuable conceptual qualities [41, 51] This methodological choice led us to try to limit evaluation bias as much as possible by designing a real “attention control group” (previously described). We also created an intervention nonspecifically designed to reduce the burden—known to influence proxy-rated QOL—but to preferentially improve knowledge and skills.

Furthermore, we thought this choice would also reflect our ethical position of a patient considered a “subject” of care rather than an “object” of care. Indeed, regarding secondary outcomes, our negative results are in accordance with those of the literature, with educational interventions found to have negative effects on the patients’ cognition [19], autonomy [18] and mood [17]. We did not find any effect on the caregiver burden, anxiety or depression, whereas the literature generally reports positive effects [6, 7, 10, 12, 22]

Nevertheless, our results are balanced since while there was no improvement in the patient’s QOL proxy-rated, the self-rated QOL was significantly increased by TPE. We will first discuss the negative result of the QOL proxy-rated and then the positive results of the self-reported QOL.

Many reasons can be given to explain the negative result on proxy-rated QOL. First, regarding the characteristics of our population of AD patients, we observed that, as reported in the literature, patients assessed their QOL as higher than the assessment by their caregivers [45, 50, 52, 55], and characteristics known to influence caregiver-reported QOL were found in THERAD (loss of functional independence [50], depression [56, 41] and apathy [52]). The level of BPSD was also relatively low in the patients (mean NPI 21), although this type of intervention is effective for BPSD [14], and BPSD is known to negatively impact (proxy or self-reported) QOL [52]. A sample of more severely affected patients (higher BPSD) may have been more pertinent to measure the impact. Our population sample in terms of the severity of the disease may also not be homogeneous enough. Indeed, the determinants of QOL in AD are different between stages; QOL is related to cognitive function during the mild stage and autonomy in moderate to moderately severe stages in the literature [50, 58, 59]. However, the internal validity of the QOL-AD scale from mild to moderately severe stages of the disease has been formally validated, allowed us to pursue this goal [35].

Regarding the intervention itself, although we created an educational intervention close to the effective multicomponent strategies in terms of content and duration, and assessed it a qualitative manner (fulfilment of educational objectives on visual analogue scale), the dyadic perspective remained challenging. Indeed we tried to help each dyad reach their own individual goals through the acquirement by the caregiver of knowledge and skills. At the end of the programme, caregivers were asked to rate their goals as “achieved” or “to reach” and to estimate skills as “acquired” or “to strengthen” on visual analogue scales. If the educational intervention can reinforce the carer in his or her role and bring about behavioural changes, then measuring any change in the way he or she provides care and, consequently, the potential impact on patient health is complex.

Moreover, the intervention needs to be standardized but also sufficiently tailored to dyadic issues. During the trial, we observed a number of issues between spouse and child caregivers that are known to be different [60]. We noted that an intervention such as ours designed to provide knowledge and skills could be more effective for spouses than child caregivers (the latter may benefit from interventions designed to alleviate the mental load and burden), as suggested in the literature [9]. It should be noted that the programme was of short duration and the intensity was quite low, while TPE is a continuous process that should be continued and adjusted to the disease course and patient lifestyle. However, previous studies assessing interventions of longer durations (3–12 months) were negative [18, 19].

Another interpretation of our contrasting results is possible since self-reported patients’ QOL was significantly increased by TPE. Indeed, we cannot exclude that the intervention may have had a positive effect on the patient that was not perceived by his or her caregiver. We hypothesize that the self-rated QOL is closer to reality in the THERAD population. Indeed, the positive significant effect on self-reported QOL at 2 months and 6 months does not remain at 12 months, whereas self-reported QOL is stable in the literature [50, 53] in this specific population of patients between mild and moderately severe AD, suggesting a possible early and time-limited effect. This stability described in the literature [44] has been attributed to the patient’s reduced abilities to estimate any change on a scale [50, 53] and, for some authors, to the conceptual nature of certain items (self-esteem) not being understood and being responsible for the missing data beyond the mild stages. Moreover, a decline in the patient’s QOL during the mild stages is related to cognitive impairment [50, 58, 59], as previously note, on which educational interventions have not demonstrated any impact [19]. Last, our subgroup analyses reported an increased intervention effect in the subgroup of moderately impaired patients, with an MMSE score of 15–20.

As mentioned, many reasons can explain this difference between proxy-reported and self-reported patient QOL. First, most of the included patients were cared for by their child, which has been reported to be associated with a worse QOL than those cared for by their spouses [45, 50]. Then, in THERAD, factors that negatively influence proxy reporting, e.g. burden [52, 55], were present, with 70% of caregivers having a moderate or high burden ZBI score > 20 (mean = 30.9)). However, burden is not alleviated by the intervention (it should be mentioned that our intervention was designed to improve caregivers’ knowledge, which is known to enhance patient QOL [61], and not to lessen the caregiver’s burden, which introduces discrepancies in QOL assessment [52]). We did not specifically provide formal psychological support. Subgroup analyses showed an improvement in caregiver-reported patient QOL among the most exhausted caregivers, particularly at M12, without, surprisingly, any reduced burden, suggesting a potential effect among exhausted caregivers [52].

## Limitations

From a methodological point of view, THERAD has several *limitations*. The recruitment setting (ambulatory units) induces a selection bias because of a “restraint” profile of AD patients (severity, autonomy, etc.) and negatively influences the generalizability of the results. However, the randomization secondarily limits this phenomenon. The monocentric design of the study is also a factor limiting generalizability of the results.

Then, as suggested by the improvement in the patients’ QOL in both groups, which is not common [50, 53], we probably provided a certain level of unintentional support in the control group despite our effort to limit this bias by designing a real “attention control group”. We can imagine our results would have been positive for proxy-rated QOL or more significant for self-rated QOL if we did not pay any attention to the control group (but in this case, it would have been difficult to disentangle attention from the intervention effect). Moreover, even if we tried to offer purely educational information, “informal psychological support” may have occurred during coffee breaks when caregivers shared their caregiving experiences with their counterparts.

Regarding the assessment of the intervention effect, a scale of knowledge [8] or a sense of competence [62] could have been used instead of QOL, despite often previously studied, because embracing all of the dimensions of one’s QOL in a formalized, reproducible but tailored format [28] was perhaps too ambitious. However, it seemed reachable in the existing literature at the time we designed the trial. Moreover, some reviews, conducted in other chronic conditions, showed that improving the caregiver’s knowledge does not necessarily lead to a change in the way help and care are provided [63].

Last, joint sessions (helpful with regard to communication skills and social abilities) would have been of interest. They will be implemented in our future TPE programme.

However, THERAD presented *several strengths*.

First, the methodological choice of a randomized controlled trial with an attention control group in such an intervention seems robust. We performed intention-to-treat analysis and per-protocol analysis to approach the theoretical effect of our intervention, and we need to emphasize that they produced comparable conclusions. The compliance was good (defined as at least 3 group sessions and the two individual sessions), and in such an educational intervention, especially with a condensed content and short duration, missing one single session was considered as missing a significant “dose” of the intervention.

Then, we used a patient-centred, clinically meaningful, relevant outcome, QOL, rather than an intermediate outcome, such as a process indicator (coping, self-efficacy), thus avoiding previously studied outcomes (burden or caregiver’s knowledge and sense of competence [8]).

The patient was fully included in the educational part. We consider our dyadic approach both in the intervention (inclusion of patients in the educational programme) and evaluation (the two perspectives) to be a strength.

As a considerable strength, such approaches will help in the fight against stereotypes [46] and encourage social inclusion.

Additional studies targeting a subpopulation of caregivers (spouse vs. child, male caregivers vs. female caregivers) and also AD patients (severity of cognitive impairment and BPSD) are necessary using the methodology of an RCT with an attention control group to both limit bias and apprehend the wealth of the two members of the dyad point of view.

## Conclusion

THERAD introduces a double perspective, by proxy and self-reporting, in the assessment of an educational intervention targeting the dyad in AD. Our contrasting results on the patient’s QOL underline the challenging issue of measuring the AD patients’ QOL due to documented bias and the need to find a robust methodological approach. THERAD also suggests the need to design educational interventions targeting subpopulations of caregivers (spouse vs. child, male caregivers vs. female caregivers) and also patients (by the severity of cognitive impairment and BPSD). More generally, attention payed to the AD patient’s point of view, introduced into dyadic approaches, is fundamental with social participation and “inclusion” being part of “living well with dementia” [46].

## Data Availability

The datasets used and/or analysed during the current study are available from the corresponding author on reasonable request.

## Abbreviations

AD: Alzheimer’s disease
ADL: Activities of daily living
BPSD: Behavioural and psychological symptoms of dementia
CGA: Comprehensive geriatric assessment
GDS: Geriatric Depression Scale
IADL: Instrumental activities of daily living
ITT: Intention-to-treat analysis
MMSE: Mini-Mental Score Examination
M0: Baseline visit
M2: 2nd month visit
M6: 6th month visit
M12: 12th month visit
NHP: Nottingham Health Profile
NPI: NeuroPsychiatric Inventory
QOL-AD: Logsdon Quality of Life in Alzheimer’s disease
TPE: Therapeutic Patient Education
TUH: Toulouse University Hospital
ZBI: Zarit Burden Interview
